# Item Roles Explored in a Modified P300-Based CTP Concealed Information Test

**DOI:** 10.1007/s10484-019-09430-6

**Published:** 2019-04-09

**Authors:** Gáspár Lukács, Alicja Grządziel, Marleen Kempkes, Ulrich Ansorge

**Affiliations:** 1 Department of Basic Psychological Research and Research Methods, Faculty of Psychology, University of Vienna, Liebiggasse 5, A-1010 Vienna, Austria; 2 Institute of Philosophy, Universtiy of Vienna, Universitätsstraße 7, A-1010 Vienna, Austria

**Keywords:** Concealed Information Test, EEG, P300, Complex Trial Protocol, Association, Saliency

## Abstract

In this study, we introduced familiarity-related inducer items (expressions referring to the participant’s self-related, familiar details: “mine,” “familiar”; and expressions referring to other, unfamiliar details, e.g., “other,” “irrelevant”) to the Complex Trial Protocol version of the P300-based Concealed Information Test (CIT), at the same time using different item categories with various levels of personal importance to the participants (forenames, birthdays, favorite animals). The inclusion of inducers did not significantly improve the overall efficiency of the method as we would have expected considering that these inducers should increase awareness of the denial of the recognition of the probes (the true details of the participants), and hence the subjective saliency of the items (Lukács in J Appl Res Mem Cognit, 6:283–284, 2017a). This may be explained by the visual similarity of inducers to the probe and irrelevant items and the consequent distracting influence of inducers on probe-task performance. On the other hand, the CIT effect (probe-irrelevant P300 differences) was always lower for less personally important (low-salient) and higher for more personally important (high-salient) items.

## Introduction

Reliable and valid deception detection methods are widely needed, for example, in criminal proceedings and for issues of public security, because without such aids it is extremely difficult — if not impossible — to tell whether a (potential) perpetrator is telling the truth or lying (Bond and DePaulo 2006; Hartwig and Bond 2011).

One of the most successful methods under development is the P300-based concealed information detection. It is based on analyzing neural activity recorded by electroencephalography (EEG). In an EEG examination, electrodes are placed on the scalp, through which electrical activity inside the brain can be detected. The P300 is the component of an event-related potential (ERP) with a positive peak arising most prominently above the parietal lobe, beginning usually around 300 ms after stimulus onset (for a review, see Polich 2007; also Donchin and Coles 1988; Johnson 1986, 1988, 1993). It is typically obtained through the “oddball” paradigm: When presenting a regular sequence of predictable stimuli, an infrequent deviating stimulus will evoke the P300 component if the oddball is uniquely different from the other stimuli; for example, when it is task-relevant (e.g., a target among a sequence of non-targets) or in any respect perceptually salient as irregular in comparison with the rest of the stimuli. Importantly, the probability with which a stimulus occurs robustly influences the magnitude of the P300: Infrequent salient stimuli evoke larger P300 components – an effect which is considered to reflect the involvement of limited-capacity cognitive processes in the generation of the P300 (Polich 2007). According to the influential context-updating theory of the P300 (Donchin 1981; Donchin and Coles 1988), this waveform represents the updating of stimulus representations in working memory, a process that is highly context-dependent, influenced by both immediate stimulus history and task demands (previous knowledge, expectation, selective attention, etc.). More recent theories emphasize the reactivation of previously established stimulus–response associations; a process that also depends on stimulus frequency (Verleger et al. 2014, 2015).

The P300 can be used in memory detection, too: in the Concealed Information Test (CIT), a deception detection method that is based on the recognition of a certain stimulus, for example, crime-relevant information, among other, irrelevant stimuli (Lykken 1959; Verschuere and Meijer 2014). For example, various items, any of which could be the murder weapon, are sequentially presented to a murder suspect: “gun,” “knife,” “rope,” etc. Here, the true murder weapon with which the actual crime was committed is the *probe* item. All other items are conventionally called *irrelevant* items (or irrelevants). The number of different irrelevants is typically around five, and each item (including the probe) is repeated 30–40 times, presented in a random order. It is assumed that the suspect will recognize the true murder weapon only if he/she has participated in the murder. The recognition of the true murder weapon as, in this respect, a semantically salient unique item will result in a larger average P300, which is statistically differentiable from the P300 elicited by the other, irrelevant items.

### Introducing Familiarity-Related Inducers to the P300-Based CIT

Initial P300-based CIT methods included one randomly designated target item, to which a different behavioral response (keypress) had to be given when it appeared, presented among the irrelevants. However, Rosenfeld et al. (2008) reasoned that this task drains processing resources, diverting attention from the recognition of the probe item, and thus also reducing the P300 response to it. Therefore, the Complex Trial Protocol (CTP) was devised so that the probe and irrelevants all required the same response: a keypress indicating merely that the participant saw the displayed item (Rosenfeld et al. 2008, pp. 906–907). Additionally, to hold attention throughout the task, after each trial of displaying a probe or an irrelevant, a simple secondary decision task was presented, with a rare target item requiring a keypress different from the one to the non-target items. In most of the following studies, this decision task involved strings of five identical numbers where the string of *11111* was the target, and strings of four other numbers (*22222*, *33333*, *44444*, and *55555*) were non-targets.

We here posit that this secondary task may have more importance than simply just holding attention. The guilty examinee in any CIT task understands that the probe item is a rare and distinct item among the irrelevants, and therefore involuntarily sees it as a *target*-*like* item. Same as the explicit targets in the initial simple CIT task, the CTP CIT secondary task of classifying number strings with a smaller proportion of target number strings may reinforce the notion of having distinct, rare target items in the task, which need to be looked out for and require a different response (see Rosenfeld et al. 2006; Lukács et al. 2017a). Since this secondary task is implemented as a fixedly alternating subtask with distinctly different item types (numbers) and primary and secondary task can therefore be nicely discriminated from one another, the secondary task may not divert attention away from the probes like the target in the original CIT task. Nonetheless, same as the target in the original task, this parallel target-nontarget task could still foster an increased awareness of the target-like nature of the probe (its importance and low frequency), which results in increased psychophysiological responses to it.

In the current experiment, we investigated if further characteristics of the secondary task could be used to increase the probe’s perceived relevance in the primary task. Following the analogous reaction time (RT)-based CIT study of Lukács et al. (2017b; Lukács 2019), we included here familiarity-related items into the secondary task, in order to further increase the distinct target-like nature of the probe item for guilty examinees by increasing their awareness of the semantic context of the lie detection scenario: namely, that by pressing the response key to the main items (i.e., items of the primary task), the participant denies the familiarity of the probe item presented (Rosenfeld et al. 2012). We therefore replace target number strings with self-referring items (expressions referring to the participants self-related, familiar details: “mine,” “familiar”), and nontarget number strings with other-referring items (expressions referring to other, unfamiliar details, e.g.: “other,” “irrelevant”; see also Lukács and Ansorge 2019a, b). Since (1) the perceived difference of the probe from the irrelevants is due to its meaning, and (2) a larger perceived difference leads to larger probe-irrelevant P300 differences (Lukács et al. 2016; Marchand et al. 2013), we hypothesized that (3) increasing this perceived semantic difference by using inducer targets would also lead to larger probe-irrelevant P300 differences, thereby increasing the sensitivity of the CTP CIT.

Here, let us shortly elaborate on the three points in the previous sentence. The fact (1) that the perceived difference of the probe from the irrelevants is due to its meaning should be clear: Dozens of articles have shown that a meaningful probe (e.g., an autobiographical detail, or an object from a mock-crime) evokes a larger P300 than a probe (foil) in the control group which has no meaning for the examinee (e.g., Rosenfeld et al. 2013). The assertion (2) that a larger perceived difference between the oddball (in our case: probe) item and irrelevant items leads to larger P300 differences – in particular due to the task-relevance of the oddball – has long been demonstrated (see e.g. Johnson 1986; Picton 1992), and even directly in a P300-based CIT study (Marchand et al. 2013; see also Lukács et al. 2016). Finally, (3) effective semantic influences via inducer items correspond to semantic congruence effects in general. When two different concepts are semantically related or congruent (e.g., a positive word and a flower), they facilitate a shared classification response (e.g., a keypress shared for all positive words and flowers). This facilitation is found relative to a situation in which semantically unrelated or incongruent concepts (e.g., a negative word and a flower) require classification by a shared response as demonstrated most prominently by the famous Implicit Association Test (Greenwald et al. 1998). Translated into the CTP CIT protocol, the status of the probe as an oddball known to the participants and being thus different from the irrelevants in the primary task would be further enhanced by its semantic congruence to the familiarity-related oddball targets among the unfamiliarity-related nontargets in the secondary task.

Furthermore, Rosenfeld et al. (2012) already provided precedent in directly proving that the awareness of the semantic context of deception can be successfully manipulated in order to increase probe-irrelevant P300 differences: In their study, during the test, participants were simply presented intermittent sham feedback messages that claimed that their lies (the denial of the familiarity with the presented probe items) had been detected via their recorded brainwaves. This simple manipulation robustly increased detection rates. We reasoned that an even larger, or at least similar enhancement could be achieved with a manipulation that could increase the awareness of deception directly embedded and continually present in the task – such as our inducers.

Yet, already at this point, we want to emphasize that this manipulation may have also come at a cost. As was done in other studies, for our primary task, we used words. However, as we also used words rather than numbers in the secondary-task inducing trials, the distinction between primary and secondary task may have been blurred in our experiment. In the standard CTP, the secondary task is clearly separated from the primary task by (1) the alternating order of the two tasks (a secondary task item always follows a primary task item), and, perhaps even more importantly, (2) the distinct, immediately recognizable visual type of simple number digits in the secondary task. With the use of inducer words in the secondary task, this second factor is unintentionally eliminated, thereby making it more difficult for a participant to easily distinguish between the two tasks, as well as between the different items within the two tasks. The participant in the original task has to look out for two types of stimuli *within* the category of semantically meaningful word items: probe and irrelevant (while the rest is simple strings of numbers, clearly separated from these word items). With inducers, when the participant sees a word item, it can belong to four categories: probe, irrelevant, target, or nontarget. On the one hand, this may increase probe-irrelevant effects due to deeper processing, as participants would have to pay more attention, so as to determine the nature of the stimulus. On the other hand, this same lower distinctiveness leads to less distinct task representations, generally increased cognitive load, and more diverting of resources away from the probes in the primary task and towards the target in the secondary task (Rosenfeld et al. 2008). As we will point out further below, we addressed this issue by including a mixed-character (i.e., numbers and words) probe that was more distinct from the secondary-task word stimuli, which allowed us to separately examine these potential effects related strictly to the visual type of the items. In any case, we expected the effect of inducers to outweigh any other influences (cf. Lukács et al. 2017b), and hence increase the efficiency of the method, overall.

### Saliency and Stimulus Types

It has been repeatedly shown that the P300-based CIT is more efficient (i.e., shows higher probe-irrelevant P300 differences) when using self-related autobiographical details (e.g., birthday dates) than when using incidental details learned prior to the experiment (e.g., a stolen object from a mock-crime; Ellwanger et al. 1996; Rosenfeld et al. 2006, 2007). This could be due to the semantic saliency of the presented items: Autobiographical details may be more personally important and more rehearsed (leading to stronger memory traces), and hence the probes will be more salient, subjectively, compared to the irrelevant items. This is essentially the same reasoning as the one we presented for the potential benefits of inducers: The perceived difference of the probe from the irrelevants is due to its meaningfulness to the subject, and a larger perceived difference leads to larger probe-irrelevant P300 differences (Marchand et al. 2013). However, in a more recent study (Gamer and Berti 2012), it was argued that the difference between autobiographical and recently learned details is rather qualitative and ambiguous in view of saliency (confounded by memory type, self-relatedness, experimental context, etc.).Therefore, these authors avoided such confounds by comparing semantically central and peripheral details from the same experimental mock-crime task (e.g., a stolen CD was central, while the office where this theft happened was peripheral). In this situation, the semantic item differences did not affect the efficiency of the P300-based CIT, and the authors concluded that the P300 may be resistant to differences in semantic item saliency, as it primarily only reflects successful item recognition (i.e., that the participants recognized the probes as the relevant items in the task, regardless of their specific semantic saliency; Gamer and Berti 2012, p. 8; see also Meijer et al. 2009). However, there was no clear independent assessment of the success of the semantic saliency manipulation (i.e., the higher semantic saliency of the central details), and therefore the lack of significant difference in P300 responses may have been simply due to too small differences in semantic saliency. Finally, none of these or any related effects were ever tested using the CTP CIT.

To clearly delineate what we mean by *semantic saliency* in the present study, we can define it as a subjectively perceived importance (as also measurable via self-reported ratings) of items that otherwise belong to the same semantic dimension: For example, in our present experiment, these items were all self-related autobiographical details, and did not include any other types of potentially confounding details, such as crime-related ones recently learned in a mock-theft. The study of Gamer and Berti (2012) was similar in that they had also used items from the same semantic dimension (only crime details, while we used only autobiographical details). However, apart from our different experimental design (CTP CIT instead of regular CIT), the item categories were also different. We used item categories that were already successfully used in several recent RT-based CIT studies to manipulate the level of semantic saliency, with expected decreased probe-to-irrelevant RT differences in case of low-salient items (Kleinberg and Verschuere 2015; Lukács et al. 2017b; Verschuere et al. 2015). Here, we chose participants’ forenames as high-salient probes, and their favorite animals as low-salient probes. In addition, we used our participant’s birthday dates as probes of intermediate semantic saliency.^1^ The latter category is interesting because it allowed us to use mixed-character items as probes, consisting of both words (months) and numbers (dates), which we hypothesized to be relatively distinct from the other word items, including the words of the secondary task. We will pick up upon this latter distinction by the label *visual stimulus type* in an analysis addressing the influence of this factor further below (in the Results section).

### Slight Changes to the Original CTP Protocol

Our aim here was not a precise replication or verification of previous CTP studies – this has already been done by Lukács et al. (2016). Therefore, we took the liberty to make some small changes to the protocol.

Primarily, we reversed the order of secondary and primary tasks within each trial. In previous studies, the probe or irrelevant item came first, and then the secondary task item, with each secondary item preceded by each primary-task (probe or irrelevant) item equal times. This is due to the historical circumstance that in the first CTP CIT study, the secondary-task items consisted of the primary-task items repeated in a different color (Rosenfeld et al. 2008, pp. 907–908). This, however, is non-pertinent for subsequent studies using number strings, and the reverse order would in fact make more sense in view of a better balanced order of the sequentially presented items: The influence of a specific probe or irrelevant item on the following secondary-task item is hardly of any interest, but the preceding secondary-task item’s effect on the processing of the following probe or irrelevant item may bias the probe-irrelevant item processing difference. For example, after seeing a rare target item, participants may be primed for unique items in general and are therefore more likely to expect an upcoming probe item, or simply be more aroused and attentive, and therefore respond with a larger P300 to the subsequent primary task item, whichever item this may be (Polich 2007). Therefore, the randomization should be realized in a way that each primary-task item is preceded by each secondary-task item equal times. Nonetheless, the test is one continuous presentation of stimuli with very few breaks, and therefore the order of alternation (i.e., primary task first vs. secondary task first) presumably loses relevance as the test progresses, and it is, therefore, probably not a crucial factor.

Another change is that in the secondary task two different targets were used instead of one. We did this in order to encourage categorization based on the meaning of the inducers, as the changing target word requires more attention and deeper semantic processing. In contrast, responding to a single target throughout the test may become rather automatic and more based on simple visual differences (e.g., number of characters) as in the case of number strings. Nonetheless, to keep the targets infrequent, the target to nontarget ratio was kept at 1:4, same as in most previous studies – consequently, either target item appeared half as frequently as any of the four nontarget items. This may have increased cognitive load, counteracting the original purpose of the CTP in retaining more processing resources (as pointed out in a previous review of this manuscript by J. P. Rosenfeld), but, considering that the target to nontarget ratio remained the same, this factor is also likely negligible.

Finally, unlike in most previous studies, the time interval from the target-nontarget presentation to the probe-irrelevant presentation was slightly randomized (1500–1700 ms). This was just a basic precaution to avoid automatic responses—once again, hardly of any relevant consequence on the method’s efficiency.

Not relevant to the CIT task itself, we also changed the procedure of the attention checks: In the original version, the task was interrupted at random points, and the participants had to verbally report the last seen item. We automatized this item recall procedure so that participants would be able to select the last item on the computer screen by themselves, instead of reporting it to an experimenter (for details, see "Procedure").

These changes, separately or together, may or may not have had a slight impact on the method, but this did not relate to our research questions, which we examined with this specific version of the CTP tailored to be more suitable to our experimental design.

## Methods

### Participants

We opened 40 slots for participation in the online Laboratory Administration for Behavioral Sciences system of the University of Vienna, offering five “experimental participation” course credits, and, correspondingly, 40 psychology undergraduate students volunteered to participate in our study. All participants had normal or corrected-to-normal vision, and all signed an informed consent before beginning the experiment.

Each participant was randomly assigned to one of two conditions: *Standard* Group (CTP CIT with number strings; the slightly modified version of the original CTP CIT) or *Induced* Group (CTP CIT using inducers instead of number strings). Five participants had to be excluded: one for very low probe accuracy (only 49% correct), one for very low target accuracy in the secondary task (number strings; overall only 56% correct; or 46% for each first presentation; see "Procedure"; "Procedure" and Appendix 1), two for misunderstanding the task (one pressing the wrong key for probes, the other pressing the wrong key for both probes and irrelevants), and one for not correctly recalling the last presented item seven out of nine times (see again under Procedure). Out of the remaining participants, 19 were assigned to the Standard Group (*M*_age_ ± *SD*_age_ = 21.53 ± 1.26 years; 9 males), and 16 to the Induced Group (*M*_age_ ± *SD*_age_ = 22.25 ± 2.65 years; 8 males). With this sample size, for our main mixed analysis of variance (ANOVA) interaction (within-subject probes vs. irrelevants, between-subjects Standard vs. Induced), calculating with a medium effect size of f = .25, at alpha level .05, we obtain a reasonable power of .82 (G*Power 3.1.9.2; Faul et al. 2007).

### Procedure

Participants were informed that the task simulates a lie detection scenario, during which they should try to hide their identities. Afterwards, items were generated using a JavaScript application programmed for this purpose (freely available at https://osf.io/3cewx/– both in original German and in a similar English version, along with a manual). For probe items, participants entered their forenames (along with gender), and selected their birthdays (month and day), and favorite animals (out of a list of 112 different animal names). On submission, for each category, eight items were chosen randomly but with the closest possible character length to the given probe (depending on the list of available items), and none of them starting with the same letter (except in case of months). These putatively irrelevant items were displayed on the screen, and the participants were asked to note any (but a maximum of two per category) items that were personally meaningful to them or in any way appeared different from the rest of the items. Subsequently, five irrelevants for the task were randomly selected from the non-chosen items (as this assures that the irrelevants were indeed irrelevant). Thus, in either condition, there were altogether 18 unique items: three probes and 15 irrelevants. These items were then copied into a Python script^2^ in PsychoPy (Peirce 2007), in which the CIT task was run.

Stimuli were presented in a 75 cm distance from the eye of the participant, on a 20 inch CRT screen. All presented characters were white on black background, with a height subtending a visual angle of approximately 0.76°.

**Fig. 1:**
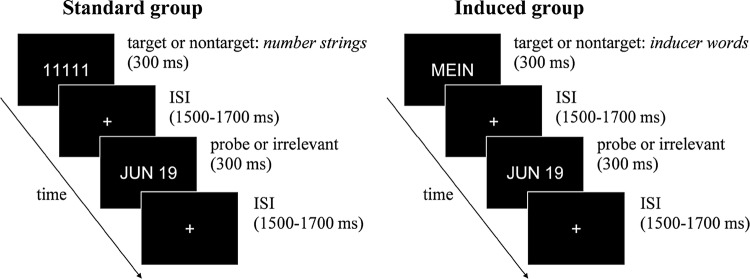
Example of a trial in the CTP CIT task in this study. The successive secondary and primary task items, and the inter-stimulus intervals (ISIs) in-between. Note that the only difference between the two versions is that, in the target-nontarget discrimination task, number strings are displayed in the Standard version, while inducer words are displayed in the Induced version. In response to targets (designated target number strings or self-referring inducers), a key had to be pushed by the right middle finger, while in case of a nontarget, another key had to be pushed by the right index finger. This was followed by the primary task’s probe (participants’ forename, birthday, or favorite animal, in separate blocks) or irrelevant items (other forenames, dates, or animals), all of which always required the same keypress with the left hand index finger. “MEIN” is German for “mine”. The arrow depicts the flow of time

Each trial began with a 100 ms baseline period for the recording of prestimulus brain activity. Then one of the secondary task target or nontarget items was presented in the middle of the screen for 300 ms. Following an inter-stimulus interval that randomly varied between 1500–1700 ms, one of the probe or irrelevant items (for the primary task) was presented for 300 ms. The next trial began after another randomly varying interval of 1500–1700 ms. During all intervals between stimuli, a fixation cross was presented in the middle of the screen. For a schematic depiction, see Fig. 1.

The primary task consisted of three blocks, with each block for one category (forenames, dates, or animals). The order of blocks was counterbalanced within each group. In each block, there were 180 trials in total, with each of the six items (one probe, five irrelevants) repeated 30 times, for a total of 30 probes and 150 irrelevants presented in a random sequence, preceded by any of the secondary-task items equal times (except for the two variations of the target, which were each half as frequent as the nontarget items; see next paragraph). The randomization was further restricted in that item-groups containing all six primary-task items (in random order) were presented successively, and the same primary- or secondary-task items in one trial never appeared in the subsequent trial.

In the Standard Group, nontarget items were four different number strings (“11111,” “22222,” “33333,” and “44444”), and target items were two different number strings (“‘88888” and “99999”). In the Induced Group, nontarget items were four different other-referring words (“ANDERE,” “SONSTIGES,” “FREMD,” and “UNBEDEUTEND”; i.e., “other,” “miscellaneous,” “foreign,” and “irrelevant”), and target items were two different self-referring words (“MEIN” and “VERTRAUT”, i.e., “mine” and “familiar”). The target to nontarget ratio was kept at 1:4, same as in most previous studies – consequently, either target item appeared half as frequently as any of the four nontarget items.

Before the main task, all participants completed a short practice task of 70–100 trials using Greek letter names as temporary primary-task items (to avoid any potential habituation bias in case of using any of the real primary-task categories for practice). In the first phase, 30 trials were presented with a response deadline of 2 s, and each incorrect or too slow response resulted in corresponding feedback (“False!” or “Too slow!” [“Falsch!” or “Zu langsam!”] in red) for 500 ms. As also in the rest of the test, in case of an incorrect response for a secondary-task item, the item was always displayed again, until the correct response was given. (This ensured that each primary-task item is preceded by a secondary-task item that was correctly responded to.) The next 30 trials had a response deadline of 1400 ms. In case of less than 85% accuracy of responses to primary-task items or to secondary-task nontarget items, or less than 75% to secondary-task target items, corresponding feedback was given and ten more practice trials followed. This was repeated maximally two more times. After reaching sufficient accuracy or after a maximum of 90 trials, 10 final practice trials were presented with no feedback in case of an incorrect or too slow response. The following data-collection phase had the exact same structure (with no feedback), only the Greek letter names were replaced with the selected probe and irrelevant items in the respective categories for each block (forenames, dates, and animals).

Throughout the task, at each appearance of any probe or irrelevant item, a key with the left index finger had to be pressed (*caret* [^] in the upper left corner of a standard German flat keyboard). In the secondary task, one of two keys had to be pushed with the right hand; one with the middle finger (*hyphen*-*minus* [-] in the upper right numpad corner) for targets, and the other one with the index finger (*asterisk* [*] left of the hyphen-minus) for nontargets.

Participants were informed that from time to time during the experiment there would be a pause and they would need to recall the last presented item. This was actually asked nine times during the test at random time points (but same for each participant), three times within each category, with a screen embedded in the experimental script: In previous studies, participants simply reported these items out loud to the experiment leader, but, to avoid such interruptions, we automatized this check: Options were displayed for all possible items in the category, indexed with the letters (a, b, c, d, e, and f), and required the participant to press the key corresponding to the letter next to the last item presented. Only one of the participants had more than four incorrect answers, and this participant was excluded (see "Participants").

After the test, there was a short questionnaire in which participants rated the personal importance of the items used in the task (their forename, birthday, and favorite animal; on a scale from one to six, where one is “entirely unimportant” and six is “very important”), and finally the participants were given a brief explanation about the purpose of the study.

### Electrophysiological Recordings and Data Processing

A DC-EEG system (neuroConn GmbH, Ilmenau, Germany) was used with electrodes recording at standard scalp sites Pz, Cz, Fz, left and right mastoids (TP9 and TP10), and four EOG electrodes (two horizontal and two vertical), with a forehead (Fpz) ground electrode, referenced online to average. The data was collected at a sampling rate of 1000 Hz, without any frequency filters.

The EEG data were processed with the EEGLAB toolbox (Delorme and Makeig 2004) for Matlab (MathWorks Inc., Natick, USA). After changing the sampling rate to 250 Hz, the data was filtered with a Hamming-windowed sinc FIR bandpass filter set to 0.3 and 30 Hz cutoff frequencies, attenuated at − 6 dB (Widmann and Schröger 2012; Widmann et al. 2015). Epochs starting at 100 ms before stimulus onset, and ending at 1400 ms after stimulus onset, were extracted, with baseline correction based on the average of the data from −100 ms to 0 ms. Ocular artifacts were then removed using an adaptive Conventional Recursive Least Squares algorithm based on the four EOG regression channels (Automatic Artifact Removal toolbox for MATLAB; Gómez-Herrero et al. 2006; Islam et al. 2016).^3^ Epochs were then rejected with amplitude over ± 75 μV at the critical Pz or either mastoid channel (leaving *M *± *SD* = 27.55 ± 2.89 epochs for each presented probe or irrelevant item in each item category for each participant), and finally the Pz was rereferenced to linked mastoids. Only this referenced Pz channel was used for all subsequent analyses.

The description of the related individual bootstrapping measure typically used for diagnostic purposes (Rosenfeld 2011; Wasserman and Bockenholt 1989) and corresponding illustrative classification rates are reported in Appendix 2.

### The Peak-to-Peak P300 Measure

A peak-to-peak P300 measure has been used in all relevant P300-based CIT studies (Rosenfeld 2011; Rosenfeld et al. 2008; Soskins et al. 2001): In our case, an algorithm searched, on the averaged epoch of a certain stimulus type, for the maximum average 100 ms segment between 400 and 800 ms, and then, between the midpoint of this segment and 1400 ms, searched again for a minimum average 100 ms segment. The choice of the search window was based on visually inspecting the grand average of all participants, verifying that the P300 peak fell within the specified window (Keil et al. 2014; Rosenfeld et al. 2015). The resulting value is the amplitude value of the peak-to-peak P300, which will be referred to as *P300* *pp* in the rest of this paper.

### Data Analysis

For all analyses of behavioral data (keypresses), responses faster than 150 ms were excluded. For all RT and P300 pp analyses, only trials with correct responses were used. Accuracy was calculated as the number of correct responses divided by the number of all valid trials (i.e., those with RT larger than 150 ms).

For all measures, following the ANOVA test for the overall Item Type (probe vs. irrelevant) × Group (Standard vs. Induced) interaction and main effects, for simplicity and clarity, all further tests for interactions (across item categories and groups) use one probe-irrelevant difference value instead of including probe and irrelevant results separately (e.g., RT_diff_ = RT_probe_ – RT_irrelevant_ for the mean RT measure; as in, e.g., Lukács et al. 2017b). Note that separate inclusion of probe and irrelevant values gives identical results.

Greenhouse–Geisser epsilon (ε) correction and corrected *p* values are reported for each *F*-test involving more than one degree of freedom. In order to demonstrate the magnitude of the observed effects, partial eta-squared (η_p_^2^) values are also shown. We also report Cohen’s *d* values for probe-irrelevant differences, following the formula given in recent RT-based CIT studies (Verschuere et al. 2015; adopted from Lakens 2013). This facilitates comparison between studies, as well as providing very good estimates for potential diagnostic efficiency.

To additionally support the lack of significant differences, we report Bayes factor values along with the *F*-tests (using the default r-scale of 0.707; Morey and Rouder 2018). The Bayes factor is a ratio between the likelihood of the data fitting under the null hypothesis and the likelihood of fitting under the alternative hypothesis (Jarosz and Wiley 2014; Wagenmakers 2007). For example, a Bayes factors of 3 means that the obtained data is three times as likely to be observed if the alternative hypothesis is true, while a Bayes factors of 0.5 means that the obtained data is twice as likely to be observed if the null hypothesis is true. Here, for more readily interpretable numbers, we denote Bayes factors as BF_1_ for supporting alternative hypothesis, and as BF_0_ for supporting null hypothesis, and we report inverse values for BF_0_ (which are originally always below 1). Thus, for example, BF_0_ = 2 again means that the obtained data is twice as likely to be observed if the null hypothesis is true.

We used the conventional alpha level of .05 for all statistical significance tests.

## Results

All behavioral and EEG data (both raw and processed) for the experiment can be retrieved from the Open Science Framework data repository via https://osf.io/3cewx/ (Foster and Deardorff 2017).

### Probe Saliency Manipulation Check

The ratings of personal importance showed the expected differences (Kleinberg and Verschuere 2015): Both forenames (*M*_rating_ ± *SD*_rating_ = 5.57 ± 1.00) and birthdays (*M*_rating_ ± *SD*_rating_ = 4.34 ± 1.00) were more semantically salient—that is, reported as more personally relevant than favorite animals (*M*_rating_ ± *SD*_rating_ = 3.34 ± 1.21), *t*(34) = 7.11, *p *< .001; *t*(34) = 3.91, *p *< .001. In addition, here we also compared ratings for forenames and birthdays: Ratings for forenames were significantly higher than for birthdays, *t*(34) = 4.16, *p *< .001.

### Behavioral Measures: RT Means and Accuracy Rates

Means and *SD*s of individual RT means and accuracy rates, along with P300 pp amplitudes, for the different trial types in either group, are given in Table 1.

**Table 1:** Reaction Time (RT) Means, Accuracy Rates, P300 Amplitudes, and Cohen’s ds, in Each Group

	Means (ms)		Accuracies (%)		P300 (μV)	
	Standard	Induced	Standard	Induced	Standard	Induced
Probe	424 ± 93	383 ± 89	97.3 ± 6.2	97.5 ± 3.7	9.65 ± 4.99	9.44 ± 4.17
Irrelevant	422 ± 96	371 ± 85	97.7 ± 3.5	97.8 ± 2.4	4.84 ± 3.12	3.79 ± 1.83
Target	516 ± 81	541 ± 72	81.6 ± 10.0	81.1 ± 9.1	13.10 ± 5.41	13.12 ± 4.84
Nontarget	469 ± 66	509 ± 77	97.4 ± 3.3	97.9 ± 1.3	6.44 ± 3.23	7.13 ± 3.49
*d*_*within*_	0.08	0.75	−0.11	−0.21	1.63	2.03

Means and *SD*s (as *M *± *SD*) for individual mean RTs, accuracies (percentages of correct responses), and P300 pp amplitudes, for *Probe* (participant’s actual self-related detail in the primary task), *Irrelevant* (other details in the primary task), *Target* (infrequent target item in the secondary task; number string [Standard group] or self-referring inducer word [Induced group]), *Nontarget* (nontarget item in the secondary task; number string [Standard group] or other-referring inducer word [Induced group]). Cohen’s *d* effect sizes as *d*_*within*_ for probe-irrelevant differences in the respective columns

To examine the primary-task probe-irrelevant item type effects (with the categories of forenames, dates, animals merged together) and its possible interactions with the variable Group (i.e., task versions) for RT means, we ran a mixed-design ANOVA, with Item Type (probe vs. irrelevant) as within-subject factor and Group (Standard vs. Induced) as between-subjects factor. The main effect of Item Type was just on the border of statistical significance (indicating slower responses to probes in both groups, as expected, see Table 1), *F*(1, 33) = 4.15, *p *= .050, η_p_^2^ = .112, BF_1_ = 1.03, while neither the main effect of Group, nor the Item Type × Group interaction was significant, *F*(1, 33) = 2.20, *p *= .147, η_p_^2^ = .063, BF_0_ = 2.45, *F*(1, 33) = 2.21, *p *= .146, η_p_^2^ = .063, BF_0_ = 1.73; despite notably larger probe-irrelevant RT effects for the Induced Group (*d*_*within*_ in Table 1).

To test potential block order effects on the probe-irrelevant RT mean differences, we ran a mixed-design ANOVA, with Block Number (first, second, or third) as within-subject factor and Group (Standard vs. Induced) as between-subjects factor. There was no significant main effect of Block Number or interaction with Group (first: *M*_RT diff_ ± *SD*_RT diff_ = 2.2 ± 20.4 s; second: *M*_RT diff_ ± *SD*_RT diff_ = 9.9 ± 32.2 s; third: *M*_RT diff_ ± *SD*_RT diff_ = 8.6 ± 26.4 s), *F*(2, 66) = 0.94, ε = .980, *p *= .396, η_p_^2^ = .028, BF_0_ = 5.01, *F*(2, 66) = 1.95, *p *= .151, η_p_^2^ = .056, BF_0_ = 1.41.

To test the effects of item categories on the probe-irrelevant RT mean differences, we ran a mixed-design ANOVA, with Item Category (forename, date, or animal) as within-subject factor and Group (Standard vs. Induced) as between-subjects factor. The main effect of Item Category was significant in the expected direction (largest for forenames: *M*_RT diff_ ± *SD*_RT diff_ = 13.9 ± 26.8 s; smaller for dates: *M*_RT diff_ ± *SD*_RT diff_ = 8.4 ± 33.0 s; smallest for animals: *M*_RT diff_ ± *SD*_RT diff_ = − 1.5 ± 15.9 s), *F*(2, 66) = 3.66, ε = .944, *p *= .034, η_p_^2^ = .100, BF_1_ = 2.02, but this effect was not influenced by Group, *F*(2, 66) = 1.30, *p *= .278, η_p_^2^ = .038, BF_0_ = 2.64.

Regarding secondary-task performance, a mixed-design ANOVA, with Item Type (target vs. nontarget) as within-subject factor and Group (Standard vs. Induced) as between-subjects factor, showed the main effect of Item Type (slower responses to target), *F*(1, 33) = 47.26, *p *< .001, η_p_^2^ = .589, BF_1_ = 1.35 × 10^5^, but neither a significant main effect of Group, *F*(1, 33) = 1.93, *p *= .175, η_p_^2^ = .055, BF_0_ = 1.20, nor an Item Type × Group interaction, *F*(1, 33) = 1.71, *p *= .201, η_p_^2^ = .049, BF_0_ = 1.68.

Similarly, for accuracy rates, we ran another mixed-design ANOVA, with Item Type (probe vs. irrelevant) as within-subject factor and Group (Standard vs. Induced) as between-subjects factor. The main effects and the interaction were not significant (*p* > .4). There were also no significant effects related to block number or item category or their interactions across the two groups (*p* > .2 for all comparisons).

In the secondary task, a mixed-design ANOVA, with Item Type (target vs. nontarget) as within-subject factor and Group (Standard vs. Induced) as between-subjects factor, showed a main effect of Item Type (lower accuracy to target), *F*(1, 33) = 137.07, *p *< .001, η_p_^2^ = .806, BF_1_ = 1.23 × 10^14^, but neither a significant main effect of Group, *F*(1, 33) = .106, *p *= .747, η_p_^2^ = .003, BF_0_ = 3.53, nor an Item Type × Group interaction, *F*(1, 33) < 0.01, *p *= .991, η_p_^2^ < .001, BF_0_ = 3.00.

### Electrophysiological Measures: P300 pp Amplitudes

**Fig. 2:**
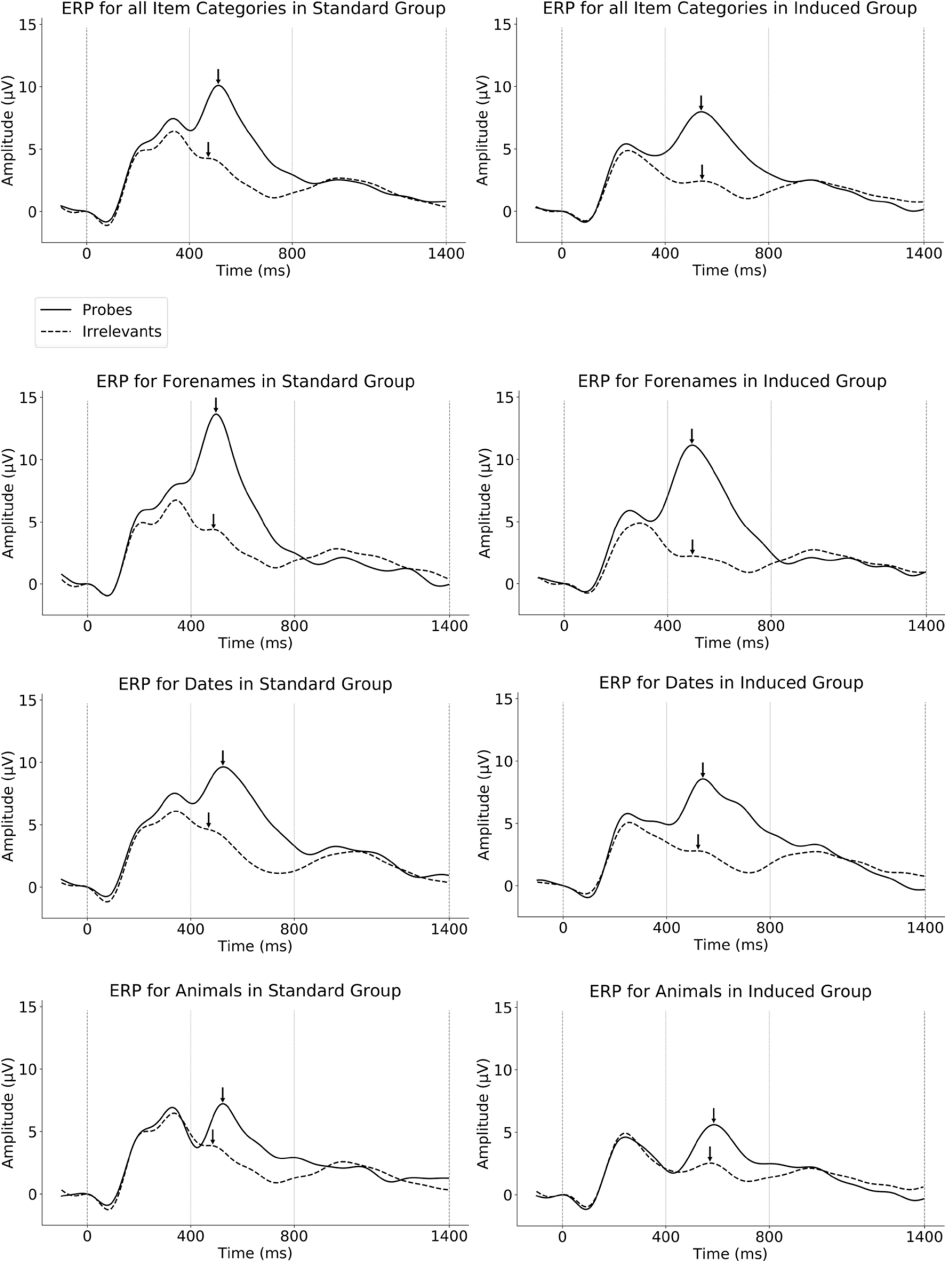
Grand average event-related brain potential (ERP) waveforms. Registered at the parietal electrode Pz, as evoked by Probe and Irrelevant items per each group, in separate panels for each item category; low-pass filtered at 6 Hz for display. Please note that while the probe P300 peak (indicated with an arrow) for the Standard version appears above that of the Induced version for all categories, the key difference is to be observed relative to the irrelevant peak (also indicated with an arrow), which is also always smaller in case of the Induced version – see also the probe-irrelevant P300 pp differences in Fig. 3

Along with the behavioral data, means and *SD*s of P300 pp amplitudes per item type and per group are given in Table 1. Furthermore, ERPs per item type, per group, per item category, are shown in Fig. 2.

To examine the primary task’s probe-irrelevant item type effect and its possible interactions across the two Groups or task versions for P300 pp amplitudes, we ran a mixed-design ANOVA, with Item Type (probe vs. irrelevant) as within-subject factor and Group (Standard vs. Induced) as between-subjects factor. We found a large main effect of Item Type (P300 pp larger for probes), *F*(1, 33) = 114.88, *p *< .001, η_p_^2^ = .777, BF_1_ = 4.44 × 10^9^, but neither the main effect of Group, nor the Item Type × Group interaction was significant, *F*(1, 33) = 0.73, *p *= .398, BF_0_ = 2.92, η_p_^2^ = .022, *F*(1, 33) = 0.29, *p *= .597, η_p_^2^ = .009, BF_0_ = 3.12.

To test potential block order effects on the probe-irrelevant P300 pp amplitude differences, we ran a mixed-design ANOVA, with Block Number (first, second, or third) as within-subject factor and Group (Standard vs. Induced) as between-subjects factor. There was no significant main effect of Block Number or interaction across the two groups (first: *M*_P300pp diff_ ± *SD*_P300pp diff_ = 6.89 ± 4.52 μV; second: *M*_P300pp diff_ ± *SD*_P300pp diff_ = 5.61 ± 4.04 μV; third: *M*_P300pp diff_ ± *SD*_P300pp diff_ = 5.89 ± 5.05 μV), *F*(2, 66) = 0.79, ε = .993, *p *= .456, η_p_^2^ = .023, BF_0_ = 5.07, *F*(2, 66) = 0.20, *p *= .816, η_p_^2^ = .006, BF_0_ = 5.49.

**Fig. 3:**
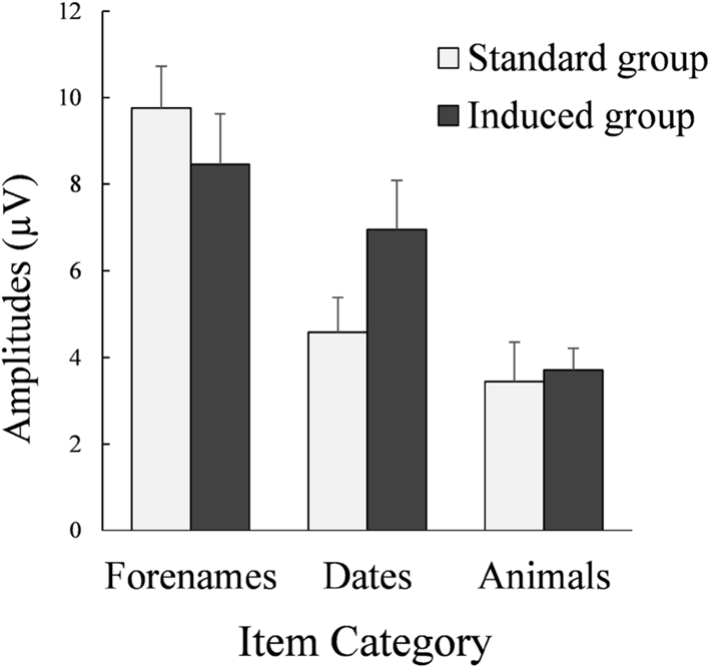
Means and *SE*s of the probe-irrelevant differences of P300 pp amplitudes. Registered at the parietal electrode Pz, for the three item categories per group

To test the effects of item categories of the probe-irrelevant P300 pp amplitude differences, we ran a mixed-design ANOVA, with Item Category (forename, date, or animal) as within-subject factor and Group (Standard vs. Induced) as between-subjects factor. The main effect of Item Category was significant in the expected direction (largest for forenames: *M*_P300pp diff_ ± *SD*_P300pp diff_ = 9.16 ± 4.43 μV; smaller for dates: *M*_P300pp diff_ ± *SD*_P300pp diff_ = 5.66 ± 4.14 μV; smallest for animals: *M*_P300pp diff_ ± *SD*_P300pp diff_ = 3.57 ± 3.17 μV), *F*(2, 66) = 29.00, ε = .990, *p *< .001, η_p_^2^ = .468, BF_1_ = 1.80 × 10^7^. This Item Category effect was also influenced by Group, as demonstrated by a significant Item Category × Group interaction, *F*(2, 66) = 3.18, *p *= .048, η_p_^2^ = .088, BF_0_ = 1.10 (see Fig. 3). (Again, there was no main effect of Group; see above.) However, follow-up *t*-tests showed no significant simple effects; for forenames, *t*(33) = − 0.86, *p *= .397, for dates, *t*(33) = 1.74, *p *= .090, or for animals, *t*(33) = 0.24, *p *= .809.

However, to explore if this interaction had something to do with the predicted stronger probe effects in trials with high versus low saliency in terms of personal relevance, we further tested potential interactions of saliency by comparing the forenames and animals (highest and lowest saliency both in ratings and in P300 pp probe-irrelevant differences) across the two groups in a mixed-design ANOVA, with Saliency (high vs. low) as within-subject factor and Group (Standard vs. Induced) as between-subjects factor. The main effect of Saliency (replicating the main effect of Item Category above) was significant, *F*(1, 33) = 60.87, *p *< .001, η_p_^2^ = .648, BF_1_ = 2.33 × 10^7^, but there was no Saliency × Group interaction, *F*(1, 33) = 1.22, *p *= .278, η_p_^2^ = .036, BF_0_ = 1.78.

This was in contrast to the influence of visual stimulus type. To test the potential interaction of the primary-task category’s visual stimulus type, we compared the P300 pp_diff_ of dates (mixed-character) and the merged average P300 pp_diff_ of forenames and animals (alphabet letters-only) across groups in a mixed-design ANOVA, with Visual Type (mixed-character vs. letter-only) as within-subject factor and Group (Standard vs. Induced) as between-subjects factor. The main effect of Visual Type was not significant, *F*(1, 33) = 0.80, *p *= .379, η_p_^2^ = .024, BF_0_ = 2.62, but the Visual Type × Group interaction was significant: compared to the Standard Version, larger probe-irrelevant P300 pp differences in the Inducer Group Version with mixed-character items, and smaller with letter-only items, *F*(1, 33) = 4.92, *p *= .033, η_p_^2^ = .130, BF_1_ = 2.39; see Fig. 3.

Regarding the secondary task, a mixed-design ANOVA, with Item Type (target vs. nontarget) as within-subject factor and Group (Standard vs. Induced) as between-subjects factor, showed the main effect of Item Type (larger P300 pp to target), *F*(1, 33) = 133.47, *p *< .001, η_p_^2^ = .775, BF_1_ = 8.50 × 10^9^, but neither a significant main effect of Group, *F*(1, 33) = 0.32, *p *= .575, η_p_^2^ = .010, BF_0_ = 3.27, nor an Item Type × Group interaction, *F*(1, 33) = 0.07, *p *= .797, η_p_^2^ = .002, BF_0_ = 2.46.

## Discussion

In the present research, we introduced familiarity-related inducer items into the CTP CIT, compared the effects of low-salient and high-salient items, and of different visual stimulus types. The inclusion of inducers did not improve the overall sensitivity of the method (probe-irrelevant P300 pp differences) as we would have expected considering that these inducers (e.g., “mine” for familiar, or “unknown” for unfamiliar) probably increase awareness of the denial of the recognition of the probe, and hence increase the subjective saliency of the probe items (Lukács et al. 2017b). Nonetheless, the probe-irrelevant P300 pp effects were lower for less personally important (low-salient) and higher for more personally important (high-salient) items as expected due to a similar reasoning, namely that the subjective saliency of the probe distinguishes it from the irrelevants, and the more the probe differs from the irrelevants, the larger the P300 differences will be (Marchand et al. 2013; Verschuere et al. 2015). While our results regarding semantic saliency are very clear, those regarding the inducers are less straightforward, and hence our explanations for the latter are somewhat speculative, serving primarily as hypotheses for further research.

It is surprising that the inclusion of inducers, which in a recent RT study literally tripled the probe-irrelevant RT mean effect sizes, showed no similar significant improvement (and, correspondingly, only very slight effect size differences) in P300 pp amplitudes. On the other hand, there are some important differences between the two methods that can explain this. Naturally, the P300 brainwave differs greatly from behavioral responses (as measured by RTs) in its mechanism. However, at the same time, as shown in this very study, the two methods both crucially depend on the saliency of the probe item, and we intended to enhance this same probe saliency via inducers in both methods. Therefore, the lack of significant improvement in case of the CTP may rather be explained by the differences in the task design.

First, unlike the standard RT-based CIT, the CTP CIT already included a secondary target-nontarget discrimination task. This may have at least to some extent functioned similarly to the familiarity-related inducers introduced to the RT task of the current study: The target-nontarget discrimination in this secondary task reinforces the notion of implicit probe-irrelevant discrimination in the primary task (see "Introduction", "Introducing familiarity-related inducers to the P300-based CIT"). While there is no reference to familiarity, the essential idea of making the participant more aware of specific target-like items in the task (i.e., the probe) is the same. Therefore, the addition of the explicit reference to familiarity may be, to some extent, redundant. (This hypothesis may be tested by replacing familiarity-related inducers in the RT task with simple target-nontarget items, such as the number strings in the standard CTP.) Secondly, this secondary target-nontarget discrimination task fixedly alternates with the primary probe-irrelevant recognition task and is not merged with the primary probe-irrelevant task. This is different from the RT-based variant of the CIT in which there was no task-switching between inducer items and probe trials. Thirdly, again unlike the RT task, the target and nontarget items in the secondary task have different response keys than the probe and irrelevant items in the primary task. Here, we kept this original design characteristic because shared response keys may have additional effects that could be worthy of a separate dedicated experiment. Shared response keys across the primary and secondary task may foster a joint representation of primary and secondary task that could again divert more resources away from the probes and towards the targets. However, it would also simplify the task in having two response alternatives instead of three, thereby reducing overall cognitive load. Moreover, it may further emphasize the denial of the recognition of the probe in classifying it with the same response key as the other-referring expressions, as opposed to the self-referring ones.

The separation in the original CTP CIT was created on purpose in order to retain more processing resources in the primary, probe-irrelevant recognition task (Rosenfeld et al. 2008). However, in our experiment, the inducer words that are, unlike simple number strings, less easy to visually distinguish from the probe and irrelevant item may have recreated the issue by reducing the distinctness of the secondary task from the primary task, and thereby causing participants to look out for the target inducer words throughout the task, decreasing attention to the probe. We hypothesized that mixed-character stimuli (dates as probe and irrelevants) could be best discriminated from the secondary-task items and, thus, are subject to least competition for resources from the targets. This was supported by our findings: The factor of visual type interacted with that of group. Compared to the standard version, effects were increased in the version with inducers in the category of mixed-character items (e.g., JUN 19), although slightly decreased when using letter-only items (e.g., MICHAEL). As noted in the Introduction (Sect. 1.2), when these letter-only word items appeared along with the inducers, participants had four item types that were all visually similar, comprising all the items in the task: probes, irrelevants, targets, and nontargets. Whenever an item appeared, its meaning had to be considered in view of these four choices, likely with a focus on the target-nontarget distinction – even in case of probe and irrelevant items – simply because this target-nontarget distinction was the explicit task requirement. However, in case of the more distinct dates, participants could focus separately on the distinction between target and nontarget inducers in the secondary task, and again separately on the distinction between probes and irrelevants in the primary task: When an item appeared, they could immediately see, due to the visual differences, whether it was a date or not, and thereafter make either a more focused target-nontarget discrimination (as explicitly required), or, most importantly, a more focused probe-irrelevant distinction (as implicitly expected).

This finding also implies a possibility to enhance the sensitivity of the method by increasing the distinctness between the primary and secondary tasks: For example, by displaying the secondary task items in lowercase or adding external marks to them (e.g., underline or frame). The specific effect of date items could also be further explored by testing the potential effect of dates as letter-only items (e.g., “JUNE NINE” vs. “JUNE 9”), or, conversely, presenting the inducers in a more visually similar format by appending random numbers to them (e.g., “MINE 19” or “OTHER 07,” as discussed by Lukács and Ansorge 2019b). However, a possible limitation is that the difference is never purely visual: The inherent circumstance that the inducers are meaningful makes them per definition semantically more similar to probe and irrelevant items regardless of appearance (e.g., in contrast to using scrambled letters or letter-only nonwords in the secondary task – which could also be tested in future research). This may still distract from the primary task more than simple semantically meaningless stimuli. Another approach then could be to enhance the original task by further simplifying the secondary task items: for example, an arrow pointing to the left or to the right, requiring response keys corresponding to the indicated direction.

As for the different levels of saliency, the same effect was shown as in RT studies; namely, higher saliency led to higher P300 pp probe-irrelevant differences. We thereby exclude the possibility that the P300-based CIT would rely merely on recognition and could be used with equal efficiency with low-salient items. This finding also further supports the notion that P300 pp does depend on subjective saliency, and therefore it should be possible to increase effects through semantic context (Rosenfeld et al. 2012) – and thus through inducers as well, when using an appropriate design.

As large differences were found between different levels of saliency, this should also be taken into account when comparing across studies using different item categories (e.g., many previous studies already used specifically either only forenames [Bowman et al. 2013; Rosenfeld et al. 2006, etc.] or only dates [Meixner et al. 2009; Sokolovsky et al. 2011, etc.]). Relatedly, there has already been a P300-based CIT study that compared items (familiar faces) with different saliency across two separate experiments (Meijer et al. 2007): However, not only item saliency, but also task instructions differed (since it was not the main aim of the study to show the effects of saliency). In one experiment, the denial of familiarity was explicit, while no deception context was given in the other. Our results would suggest that the difference between the experiments in probe-irrelevant P300 differences could potentially be attributed to saliency differences only, and not to any differences in instructions – as also corroborated by yet another study that directly compared and found no differences between probes in different tasks, displaying the CIT task as explicit deception or as simple recognition task (Kubo and Nittono 2009).

Finally, we recommend including low-salient probes in future studies as potential moderators, which would also reflect less salient items in real world applications, where there may be a scarcity of such markedly personally relevant items that are usually used in autobiographical CIT studies (e.g., personal names or hometowns).

## Appendix 1

### Further Details on Exclusions

As for exclusions based on accuracy rates, we followed recent RT-CIT studies by a limit of 50% for each of the four item types (probes, irrelevants, targets, nontargets; see Lukács et al. 2017b). However, since the secondary items were automatically replaced after an incorrect response (after which a correct response was of course much easier), for targets and nontargets we calculated accuracy for exclusion based on first responses only. (Only one related exclusion resulted, and the inclusion of this participant in the main comparisons does not change our major results; the data can be tested via https://osf.io/ts2py/.)

As for recalling the last items, typically one or two mistakes have been allowed in previous studies, but we increased this to four due to some issues with our novel automatized method for recalling the last item. The advantage of this method was simply that the participant could do the task without any interruption by having to speak with the experimenters. The disadvantage however was that they could very easily make mistakes, not by inattentiveness to the test, but by inattentiveness when pressing a key in response to this question: For example, choosing letter “C” because the item name started with letter “C,” while in the list it was, for example, by letter “D,”, hence requiring the “D” key; or quite simply missing the key in the dimly lit room—as these were also reported by some of the participants after the test. Consequently, while most participants made zero, one, or two mistakes, some of them made three, and two of them made even four mistakes—however, even these latter two demonstrated normal accuracy rates in the test and had regular P300 responses, and therefore we decided not to exclude them. One participant failed to recall seven out of nine last items. This participant was excluded.

## Appendix 2

### Individual P300 pp Bootstrapping for Classification

While diagnostic accuracy is not relevant to the research questions in our study, it may be of interest to additionally report the measure typically used for that purpose. For individual classification using P300 waves, a certain bootstrapping method has been used in all CTP CIT studies, which compares the responses to the probe item with the responses to irrelevant items. Our procedure was the following. First, single trials were chosen randomly, with replacement, from all probe single trials (i.e., trials in which the probe item had been presented), and averaged into one epoch, from which a P300 pp was calculated. The number of these chosen values was equal to the number of available probe trials in case of the given individual’s results. However, to avoid potential bias in case of oversampling a specific item (e.g., taking more single trials from a high-salient item), always equal numbers of (randomly chosen) single trials were taken from each probe item. Second, a similar number of single trials were again chosen randomly, with replacement, from all irrelevant single trials (i.e., trials in which one of the irrelevant items had been presented), and averaged into one epoch, from which another P300 pp was calculated. Again, always equal numbers of single trials were chosen for each irrelevant item, and therefore the number of items was always complemented (by maximum 14) to be divisible by the number of different irrelevants (15 items). Third, the P300 pp obtained from the probe trials was compared to the P300 pp obtained from irrelevant trials, in order to determine whether the former is greater than the latter (with a difference greater than zero). These three steps were repeated 1000 times, with results possibly varying according to the random choices with replacement. The end result of this procedure is a number between 0 and 1000, indicating the number of occasions in which the P300pp values of the probe trials were determined to be greater in comparison to those of the irrelevant trials.

The average of individual bootstrap results for the Standard Group was 936.7 ± 139.4 (as *M* ± *SD*), while it was 990.3 ± 28.4 for the Induced Group. All values per subject are available via https://osf.io/3cewx/.

To classify a participant as guilty, a specific cutoff for the bootstrapping measure is used that typically varies between 80–90% (Rosenfeld et al. 2013, p. 13). For example, if the cutoff is 90%, when the bootstrap result for the individual is a number larger than 900 (i.e., the P300pp values of the probe trials were determined to be greater in more than 900 out of the 1000 calculations), then the participant is classified as guilty. Importantly, the optimal cutoff varies by study: For example, in the study of Lukács et al. (2016), these values for guilty participants were lower than those typical in the previous studies (conducted in the lab of J. P. Rosenfeld). However, the values were also lower for innocent participants, and hence the discrimination between guilty and innocent was just as high as in previous studies, albeit using a lower cutoff value. This means that the specific task design or the equipment may have differentiated less between the responses elicited by different stimuli – however, since this affected all conditions equally, it did not affect the overall efficiency of the method. In conclusion, our calculated ratios of correct detections are only illustrative approximations: Using the arbitrary but typical cutoff rates of a stricter 90% or a more liberal 80%, we can correctly evaluate 15 or 17, respectively, out of 19 participants as guilty (78.9% or 89.5% correct) in the standard group, and 15 or 16 out of 16 (93.8% or 100% correct) in the induced group. These discrimination accuracies approximately correspond to those in previous studies, notwithstanding our inclusion of low-salient probes (favorite animals).

## Footnotes

1 Note that the category of dates has been shown to be more salient than favorite animals in RT-based CIT studies (e.g., Verschuere et al. 2015), but we also presumed that they are less salient than forenames, hence providing a midway category.

2 Available upon request.

3 The ocular artifacts from one of the datasets (for a participant in the Induced group) were removed using independent component analysis (ICA; Hyvärinen and Oja 2000) implemented in EEGLAB, because of partially missing EOG channel recording due to a technical mistake. This method separated independent subcomponents of the EEG, among which those associated with eye movements were identified on the basis of visual inspection of their single-trial activations and scalp topography and rejected. The P300 measures obtained from this dataset were typical of the group, and the exclusion of this data would not alter any of our main results.
